# Video-Guided Optimization of Stimulation Settings in Patients with Parkinson’s Disease and Deep Brain Stimulation

**DOI:** 10.3390/brainsci14090914

**Published:** 2024-09-11

**Authors:** Hannah Jergas, Julia K. Steffen, Charlotte Schedlich-Teufer, Joshua N. Strelow, Johanna Kramme, Gereon R. Fink, Veerle Visser-Vandewalle, Michael T. Barbe, Jochen Wirths

**Affiliations:** 1 Department of Neurology, Faculty of Medicine, University Hospital Cologne, University of Cologne, Kerpener Straße 62, 50924 Cologne, Germany; hannah.jergas@uk-koeln.de (H.J.); julia.steffen@uk-koeln.de (J.K.S.); charlotte.schedlich-teufer@uk-koeln.de (C.S.-T.); joshua.strelow@web.de (J.N.S.); gereon.fink@uk-koeln.de (G.R.F.); 2 Department of Functional Neurosurgery and Stereotaxy, Faculty of Medicine, University Hospital Cologne, University of Cologne, Kerpener Straße 62, 50924 Cologne, Germany; johanna.kramme@uk-koeln.de (J.K.); veerle.visser-vandewalle@uk-koeln.de (V.V.-V.); jochen.wirths@uk-koeln.de (J.W.); 3 Institute of Neuroscience & Medicine (INM-3), Forschungszentrum Jülich, Leo-Brandt-Straße, 52425 Jülich, Germany

**Keywords:** telemedicine, remote treatment, home-based treatment

## Abstract

Deep brain stimulation (DBS) for Parkinson’s disease (PD) often necessitates frequent clinic visits for stimulation program optimization, with limited experience in remote patient management. Due to the resource-intensive nature of these procedures, we investigated a way to simplify stimulation optimization for these patients that allows for the continuous monitoring of symptoms while also reducing patient burden and travel distances. To this end, we prospectively recruited ten patients treated with DBS for PD to evaluate the feasibility of telemedicinal optimization in a home-based setting. Patients recorded daily videos of a modified Unified Parkinson’s Disease Rating Scale (UPDRS) III, which experienced DBS physicians located at the clinic assessed to provide instructions on adjusting stimulation settings using a handheld programmer with previously set programs as well as patient amplitude control. This study concluded with significant improvements in participants’ motor status as measured by the UPDRS-III (*p* = 0.0313) compared to baseline values. These findings suggest that remote video-guided optimization of DBS settings is feasible and may enhance motor outcomes for patients.

## 1. Introduction

In the light of the COVID-19 pandemic [1] and recent advances in technology [2], telemedical evaluation has become increasingly helpful when caring for patients with movement disorders [3]. In particular, for patients with motor disabilities, telemedical appointments can be more convenient than frequent visits to the clinic [4,5]. Despite some shortcomings (e.g., limited physical examination, technological issues, and data safety concerns) [6,7], telemedicine is well accepted among patients, caregivers, and their physicians and improves the patients’ motor status [4,8].

As part of the advancements in treating Parkinson’s disease, deep brain stimulation (DBS) has emerged as a crucial surgical intervention. This procedure involves implanting electrodes in specific brain regions, such as the subthalamic nucleus (STN) or globus pallidus, to deliver electrical impulses that modulate neural activity. By targeting these areas, DBS effectively alleviates motor symptoms like tremors, rigidity, and bradykinesia, especially in patients with advanced stages [9].

PD patients receiving deep brain stimulation (DBS) rely on specialized care and often require extensive visits to the clinic, as DBS device programming is usually not performed outside the implanting center. Experience in telemedical treatment for these patients is limited to date, although retrospective reports indicate feasibility [10,11,12]. However, these case series used DBS systems which can be programmed remotely but are not available in the European Union [11,12], limiting transferability, or do not report standardized outcome scores [10].

The present study focused on home-based video treatments with at least one video assessment per day. Experienced DBS physicians provided telemedical advice via telephone after assessment of the videos. Patients were asked to adjust stimulation parameters accordingly using a handheld programmer. We employed this prospective design to demonstrate that telemedical care has the potential to improve motor status as measured by a modified version of the UPDRS-III [13].

To this end, we enrolled ten patients with STN DBS. Eight participants completed the protocol. One patient dropped out due to a COVID-19 infection during the study; another patient had to be excluded due to technical difficulties. Patient data are provided in Table 1.

## 2. Materials and Methods

### 2.1. Study Procedure

We present a prospective study conducted at the University Hospital of Cologne between October 2020 and December 2021.

All study procedures followed the Declaration of Helsinki, and all patients signed written informed consent forms before participation. The local ethics board approved this study (Processing no.: 19-1136_1), and this study was registered at the German clinical trials register (No. DRKS00023586).

Patients were randomly selected from patients who had received DBS at our center and met the inclusion criteria. Prior to enrollment, each patient received a physical examination with a standardized monopolar review (MR) to assess each electrode contact and its side effect profile and determine the most effective stimulation settings [14]. This procedure is part of our center’s clinical routine three months postoperatively. Motor effects were assessed after cessation of dopaminergic agents for at least twelve hours (medication off). We examined rigidity per item 22 of the Unified Parkinson’s Disease Rating Scale (UPDRS)-III. For tremors, the mean of two UPDRS-III items was assessed (item 20, rest tremor; item 21, postural tremor). The severity of akinesia was assessed according to the mean of item 23 (finger tapping) and item 25 (hand rotation). A standardized amplitude of 2 mA was used. Side effect thresholds were assessed under regular medication by increasing the stimulation in steps of 0.5 mA until side effects occurred or a maximum amplitude of 5 mA was reached.

Inclusion criteria included the following:

- Confirmed diagnosis of idiopathic Parkinson’s syndrome (according to the MDS criteria).
- Bilateral implantation of directional electrodes for deep brain stimulation in the subthalamic nucleus.
- DBS implantation occurred at least 3 months prior.
- Monopolar review as per routine clinical care.
- Minimum age of 18 years and full ability to consent to participate in this study.
- At the time of monopolar review, patients were stimulated omnidirectionally.
- Consent to participate in this study after verbal and written explanation.

Exclusion criteria included the following:

- Monopolar review incomplete or inconclusive.
- Full-time employment or lack of time to perform video tests.

Optimized stimulation programs were designed during an inpatient stay by two experienced DBS clinicians (JW and HJ) based on information gained from the MR. The original program was preserved on the first of the four programming slots on the implanted pulse generator (IPG) for safety reasons. The second slot was used for another copy of the original program. The third slot was used for a novel program with the most effective directional contacts (according to the MR) switched on. If the MR indicated that there were two equally effective programming options, we set up another program on the remaining fourth slot. Patients were blinded to the programs and hence not aware of whether they were using their old program or a recently programmed one. Each program was fitted with patient amplitude control which can be adjusted using the handheld programmer. This tool is handed to the patients after implantations and allows them to adjust the settings of their implanted device. It does not only allow them to increase or decrease the stimulation amplitude, but patients can also switch between preset programs, turn the system on or off, and monitor the battery status.

The respective program’s upper limit was the maximum amplitude at which no side effects occurred, and the lower boundary was set 1 mA below the clinical stimulation amplitude as evaluated in the MR. Patients left the clinic with their original program on.

Within a few working days after study enrollment, a commercial provider (MVB—Medizinische Videobeobachtung GmbH, Koblenz, Germany) installed the video equipment at the patient’s home. The video recording station features a Logitech^®^ Streamcam (Logitech international S.A., Apples, Switzerland), a Samsung printer (Samsung Electronics Co., Ltd., Suwon, Republic of Korea), and a remote control designed by MVB. Clips have a length of approximately 3 min. The equipment has previously been adapted to be used specifically in patients with movement disorders, and the camera that transmits data to the clinician has sufficient resolution for proper evaluation of the patient’s motor features. The patient performs motor tasks following an adapted version of the UPDRS-III [13]. Patients rated their motor status on a six-point scale ranging from one (=excellent) to six (=worst). We asked the participants to provide the video in the morning before the first dose of dopaminergic medication was taken to ensure valid ratings unaffected by fluctuations. More videos could be recorded at the patient’s request if they wanted to demonstrate symptoms or side effects that were not present before. After one to three days in their original program, patients were randomized to any program and continued the video assessment. Randomization was carried out using a random number generator. We opted for randomization to avoid potential training effects as patients might become used to the video protocol.

**Figure 1:**
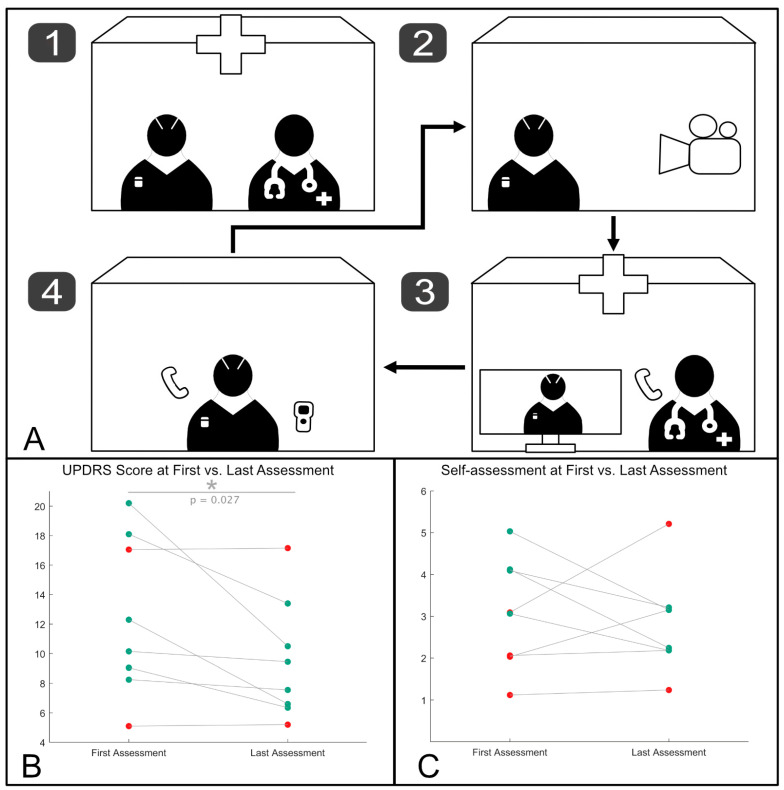
(**A**) This shows pictograms revealing the workflow during the video assessments. (1) The participants presented to the clinic, where a structured monopolar review of all effects and side effects of all contacts was conducted, and programs and patient amplitude control thresholds were set. (2) The participants recorded standardized videos at home, which were (3) evaluated by clinicians who worked on the treatment plan and the optimization of DBS settings. The DBS physicians’ recommendations were then communicated (4) to the patients via phone, who adjusted the programs using the handheld programmer. (**B**) This shows the change in motor function scores based on the modified version of the UPDRS-III between the first and last assessments. The scores range from 4 to 20, representing a subset of key motor functions evaluated during the study. Participants whose motor function improved are shown in green, while those whose condition did not improve or worsened are shown as red dots. The change between the first and last assessments was statistically significant. (**C**) This presents the patient-reported motor function scores using the German school mark system, with scores ranging from 1 (best) to 6 (worst). This figure illustrates the change in self-assessment between the first and last video assessments. Although the difference was not statistically significant, four out of eight patients reported an improvement in their motor ability.

Patients switched the programs using the handheld programmer according to instructions. Videos were rated by two DBS experts (JW and HJ). Based on the individual symptom profiles and the occurrence of side effects, patients were instructed to change the stimulation amplitude using the previously set patient amplitude control limits. If no further options for optimization remained, the participant was randomized to the next program, and optimization efforts were repeated. The exact procedure is depicted in Figure 1A. After completing optimization in the last remaining program, patients switched to their preferred setting for the last video assessment. After finalizing the video assessments, participants answered a 14-point questionnaire we adapted from [8] to evaluate tolerability and feasibility from the patient’s perspective.

Medication was not changed during the period of video assessments.

### 2.2. Statistical Analysis

Values for the modified UPDRS-III ratings (measured in points) and individual assessments of motor status on a German school mark scale for both time points (baseline and after completion of the study protocol) were compared using Wilcoxon signed-rank tests to detect statistically significant differences.

The results (Figure 1B,C) were visualized using in-house Matlab scripts. All statistical analyses were carried out in Matlab 2019 b (MathWorks, Natick, MA, USA).

## 3. Results

Participants recorded 14.9 (SD 5.98) videos on average. The total number ranged from 10 to 28 videos during the 14-day observation period.

The results of the questionnaires indicated the feasibility and acceptance of the procedure. Most participants reported that their complaints were captured better with telemedical treatment than with hospitalization (*n* = 5) or an outpatient appointment (*n* = 7). The questionnaire results are detailed further in the Supplementary Materials.

The study results are summarized in Figure 1B,C, and different scales were used to assess patient outcomes.

Figure 1B displays scores from the UPDRS-III, an objective clinical tool used to measure motor impairment in Parkinson’s disease. The UPDRS-III scores, ranging from 4 to 20 in this study, represent a subset of motor functions typically assessed, focusing on the core motor symptoms of the patient population. The participant’s mean modified UPDRS-III score during their first video assessment was 12.37 pt. (SD 5.37 pt.). At the last video assessment, scores had improved to a mean of 9.12 pt. (SD 4.12 pt.). Six out of eight patients had improved. In two patients, the UPDRS values remained unchanged. A Wilcoxon signed-rank test revealed significant improvement (T = 0.00, z = −2.207 *p* = 0.027: cf. Figure 1B).

Figure 1C presents data based on the German school mark system, a patient-friendly scoring scale ranging from 1 to 6, which was employed to gauge patients’ subjective self-assessments of their motor function. This scale was chosen for its familiarity and ease of use by patients. In self-assessment, four out of eight patients experienced that their motor ability improved. The cohort’s mean score had improved from 3 (SD 1.29) in the first video to 2.65 (SD 1.19) in the last video, although the Wilcoxon signed-rank test revealed that this difference failed to reach statistical significance (T = 7.00, z = −0.750 *p* = 0.44; cf. Figure 1C).

## 4. Discussion

The present study of PD patients with STN-DBS shows that remote assessments and video-guided home-based optimizations of DBS are feasible, generally accepted by the patients and their caregivers, and even have the potential to improve motor status.

This prospective feasibility study has several limitations. For example, our possibilities for optimizing stimulation settings were limited due to the maximum number of four programs on the DBS systems and amplitude limits. This restriction reduced the optimization options compared to inpatient visits. Further, we could not assess whether patients adjusted the programs according to our suggestions. Recently, technological advances have enabled clinicians to remotely access the patients’ IPG and change program settings through a virtual clinic platform. While approved by the FDA and commercially available in the United States and the European Union, this feature is only available for patients with specific hardware [5].

One of the patients could not complete the study protocol due to technical difficulties. The latter example underlines the need for comprehensive and accessible technology that does not bar the elderly and disabled from receiving telemedical care. This point is often critically reviewed when discussing telemedicine.

Due to the nature of this study as a proof-of-principle evaluation, we have included a limited number of patients to explore technical challenges, patient satisfaction, and the accuracy of telemedical assessments. In summary, while our data provide preliminary insights, the small sample size limits the ability to draw broad, definitive conclusions.

Another limitation of our study protocol is that the full range of side effects (e.g., slight cramps) and motor symptoms (e.g., rigidity and postural stability) cannot sufficiently be evaluated via telemedicine compared to a face-to-face evaluation. Thus, remote visits are susceptible to incomplete or faulty symptom profiles. On the other hand, telemedical assessments based on pre-recorded videos may also reveal symptoms that would otherwise go unnoticed by clinicians who only see patients at single time points. A previous study by Heldman and colleagues combined telemedical evaluation and adaptation of medication with wearable sensors that are used to objectively assess the patient’s symptoms and response to treatment [15]. A combined approach may also be useful in patients with DBS and has the potential to enable continuous and objective monitoring. Additionally, previous studies have employed deep learning algorithms in the prediction of motor status during fluctuations and medication response [16,17]; a similar application could be useful when guiding patients with DBS in a home-based setting to allow for the timely adaptation of stimulation parameters or medication during the progressive disease course.

Further evaluation of telemedicine in the treatment of DBS patients should also include symptoms that may not immediately respond to modifications of the DBS devices (e.g., dystonia or axial symptoms in PD).

Despite the discussed limitations, we conclude that our data suggest that improving motor status through telemedical care in PD patients treated with STN-DBS is feasible. Further prospective studies are warranted that include larger patient samples and directly compare telemedical versus personal appointments.

## Figures and Tables

**Table 1:** Subject information.

Subject Number	Age [y]	Sex	Disease Duration [y]	Time Since DBS Implantation [m]	Lead Type	Setting at Baseline	Setting after Study Completion
1	72	female	22	9	Cartesia, Boston Scientific	Omnidirectional	Directional
2	53	female	6	16	Cartesia, Boston Scientific	Omnidirectional	Omnidirectional
3	57	male	1	5	Cartesia, Boston Scientific	Omnidirectional	Directional
4	60	female	9	4	Cartesia, Boston Scientific	Omnidirectional	Directional
5	64	male	8	11	Cartesia, Boston Scientific	Directional	Directional
6	58	male	5	8	Cartesia, Boston Scientific	Omnidirectional	Directional
7	52	male	5	6	Cartesia, Boston Scientific	Omnidirectional	Directional
8	71	female	8	6	Sensight, Medtronic	Directional	Directional

Legend: y—years, m—months, and DBS—deep brain stimulation.

## Data Availability

The data that support the findings of this study are available from the corresponding author, M.T.B., upon reasonable request. The data are not publicly available due to privacy and ethical restrictions.

## Supplementary Materials

The following supporting information can be downloaded at https://www.mdpi.com/article/10.3390/brainsci14090914/s1, Table S1: Frequency of responses in a questionnaire as provided by eight participants and the number of videos.
