# Supplementary material for: Video-Guided Optimization of Stimulation Settings in Patients with Parkinson’s Disease and Deep Brain Stimulation
Source: Brain Sci. 2024 Sep 11;14(9):914. doi: 10.3390/brainsci14090914 (PMC11430434; doi:10.3390/brainsci14090914)
Supplement: Supplementary file 1 [file brainsci-14-00914-s001.zip › brainsci-3179744-supplementary.pdf]

Table S1. Frequency of responses in a questionnaire as provided by eight participants and number of videos.

Participant Number	*1*	*2*	*3*	*4*	*5*	*6*	*7*	*8*
Could you start the camera on your own?	Yes	Yes	Yes	Yes	Yes	Yes	Yes	Yes
Did you need assistance for any of the procedures?	No	No	No	No	No	No	No	No
Were your complaints captured better than in a hospital stay for PD?	No	Yes	Yes	Yes	Yes	No	No	Yes
Were your complaints captured better than in an ordinary outpatient visit?	No	Yes	Yes	Yes	No	Yes	Yes	Yes
Would you repeat the therapy?	Yes	Yes	Yes	Yes	Yes	Yes	Yes	Yes
Would you recommend the therapy to other PD patients?	Yes	Yes	Yes	-	Yes	Yes	Yes	Yes
Was the therapy time-consuming?	No	No	No	No	No	Yes	No	No
Did you benefit from the procedure?	Yes	Yes	Yes	Yes	Yes	Yes	Yes	Yes
Did the therapy disturb your daily routines?	No	No	No	No	No	No	No	No
Did the therapy frighten you?	No	No	No	No	No	No	No	No
Did you feel observed by the camera?	No	No	No	No	No	No	No	No
Was the procedure useful?	Yes	Yes	Yes	Yes	Yes	Yes	Yes	Yes
Could you explain your symptoms to your doctor?	Yes	Yes	Yes	Yes	Yes	Yes	No	Yes
Are you content with the therapeutic steps taken during the procedure?	Yes	Yes	Yes	Yes	Yes	Yes	Yes	Yes
Number of videos	16	14	19	13	19	10	13	28

Legend: Scheme PD—Parkinson's disease
